# Fathers’ presence and adolescents’ interpersonal relationship quality: Moderated mediation model

**DOI:** 10.3389/fpsyg.2023.1117273

**Published:** 2023-04-04

**Authors:** Ao Li, Li Sun, ShiQing Fan

**Affiliations:** Institute of Educational Sciences, Hubei University of Education, Wuhan, China

**Keywords:** parenting style, fathers’ presence, social responsibility, relationship, adolescents in China

## Abstract

**Introduction:**

Most previous studies focused on the effects of fathers’ presence on adolescent development, but rarely examined the mechanisms underlying the presence of fathers on adolescent development. Moreover, previous studies ignored the impact of fathers’ way of being present on adolescent interpersonal relationships. Based on social identity theory, the present study introduced adolescents’ social responsibility as a mediating variable to explore the influence of father’s presence style on adolescents’ interpersonal. This study examined the mechanism of fathers’ way of being present on father’s presence, adolescents’ social responsibility, and their quality of interpersonal relationships; if fathers adopt a democratic approach to be present, the study examines whether teenagers are more likely to enhance their sense of social responsibility and achieve harmonious interpersonal relationships.

**Methods:**

Participants were 1,942 senior high school and college students who responded to the Fatherhood Questionnaire, Social Responsibility Questionnaire, and Interpersonal Relationship Quality Diagnosis Scale. This study used PROCESS macro of SPSS 24.0 and Amos 26.0 to examine the hypotheses.

**Results:**

Empirical results demonstrated that (a) fathers’ presence is directly and positively related to adolescents’ social responsibility, (b) fathers’ presence is indirectly and positively related to the quality of adolescents’ interpersonal relationships through social responsibility, and (c) parenting styles played a moderating role in the first half of the fathers’ presence on social responsibility and the quality of interpersonal relationships. Results demonstrated that more harmonious interpersonal relationships were present among teenagers when fathers adopted a democratic upbringing, and this interaction effect on interpersonal relationships was mediated by teenagers’ sense of social responsibility.

**Discussion:**

The findings of this study enrich the literature by exploring the significance of emphasizing fathers’ democratic presence on teenagers’ sense of social responsibility and interpersonal relationships. The practical implications of this study are that society should encourage more fathers to be present and guide them to adopt a democratic parenting style that will benefit adolescents’ development and family well-being.

## 1. Introduction

This study investigated the effect of fathers’ way of being present on father’s presence, adolescents’ sense of social responsibility, and their quality of interpersonal relationships in China. Based on social identity theory, the present study introduced adolescents’ social responsibility as a mediating variable to explore the influence of the style of father’s presence on adolescents’ interpersonal quality. Our findings reveal that “fathers’ way of being present will influence the effect of their presence.” If fathers adopt a democratic approach to be present, teenagers are more likely to enhance their sense of social responsibility. Furthermore, fathers’ democratic presence leads to more harmonious interpersonal relationships among teenagers. The purpose of this study was to encourage more fathers to be present in their children’s lives and guide them to adopt a democratic parenting style that will benefit adolescents’ development and family harmony, happiness, and progress.

In addition, several researchers have examined the key role of fathers in children’s growth, in particular with regard to matters such as gender equality, individual mental health, children’s early academic success, less truancy, the development of children’s autonomy and moral system, etc., (Baker et al., 2018; Papaleontiou-Louca and Al Omari, 2020; Grau et al., 2022; Venta et al., 2022; Waqar et al., 2022). There have also been studies that emphasize the importance of fathers to children’s growth from the perspective of the father’s absence. For example, the absence of fathers may lead to children’s unhappiness, anxiety, poor social skills, physical illness, cognitive impairment, and depression (Billah et al., 2022; Culpin et al., 2022; Farooqui and Barolia, 2022; Gillera et al., 2022; Rodríguez Sánchez, 2022). In particular, to improve fathers’ initiative to participate in their children’s upbringing, several scholars worldwide have discussed the issue of providing or extending paternity leave (Knoester et al., 2019; Petts et al., 2020; Wray, 2020). Paternity leave is definitely critical, but it only means that fathers will be there to care for their children, and cannot guarantee the quality of father’s presence; and the quality of father’s presence is the more crucial factor for the development of teenagers.

It is worth noting that before COVID-19, various studies focused on the effects of fathers’ presence on adolescent development (Luo, 2020; Shuang, 2020), but few examined the underlying mechanisms. Subsequently, the COVID-19 pandemic forced working fathers back into the home and made their presence mandatory (Gao et al., 2021; Xiaoyi and Duan, 2022). It is extremely important to examine the impact of father presence on adolescent development. This importance is supported by the fact that different parenting styles lead to different educational outcomes. Some styles manifest in prominent family conflicts, with children and parents in a state of antagonistic clash. Others are characterized by a harmonious, happy family atmosphere, where the parents lead the children to grow up together. So what exactly is at play in the middle? Importantly, it is not only the presence of the father that is of relevance, but also the empirically best parenting style for the father to use.

Global research on fathers’ presence has advanced and risen to a new stage of “measuring and improving the quality of father-child relationships” (Stgeorge and Freeman, 2017; Palkovitz, 2019). Furthermore, given the importance of family education on fathers’ presence, since a long time ago, many policies have been introduced abroad to support family education. For example, Coleman’s report (Meier, 1967) published in the US affirmed the role of family education in children’s development and proposed that the main factor affecting children’s achievement is family, in particular, the family’s socioeconomic status (SES), with the influence of school being secondary. The 44th United Nations General Assembly (1989) passed a resolution designating 1994 as the International Year of the Family, and the United Nations Commission for Social Development (United Nations [UN], 1993) declared May 15th as the International Day for Families.

However, compared with other countries, research on fathers’ involvement in China remains at a nascent stage. The research on fathers’ presence by domestic scholars mainly presents the following characteristics: In China, the role of fathers in family education is placed in an indirect and secondary position. The father’s role often needs to rely on the mother to function (Zou et al., 2019; An and Zeng, 2022). For example, in parent-child interactions, the father’s influence on the child is mainly conveyed through the mother. Overall, the extent of research is not deep enough and there is a lack of research on the intrinsic action processes underlying fathers’ influence on adolescents. Some salient aspects of the current situation of fathers’ parenting involvement in China are as follows. First, from the perspective of society, China has followed the tradition of “men dominating the work while women dominate the house”; thus, it is a common occurrence for fathers to be at work and absent from the home (Thobejane and Florence, 2018). Chinese people lack sufficient awareness of the absence of fathers (Jiang, 2018). Second, from the perspective of family structure, the current generation of parents in contemporary Chinese families are mostly only (sole) children, owing to the former one-child policy (1979). Compared with non-only-child children, only-child children tend to be self-centered and lack independence (Rahmani and Ulu, 2021). They are accustomed to giving their children to the older generation to raise, again resulting in children lacking the company of their fathers. Finally, on the individual level, if the father is absent, the mother will take complete responsibility for educating her children. Without fathers’ assistance, unitary education of mothers may be isolated and helpless (Karunanayake et al., 2021), frequently leading to physical and mental problems among teenagers (Lee and Allen, 2021). Thus, the topic of fathers’ presence in the Chinese context is not outdated, and further research is required. In China, the individual and the mother live in the same brain region, the representation of the Chinese mother is closely related to the self, whereas the Western mother is separated from the individual self (Jing and Li, 2000). Moreover, the relationship between the child and the mother is much closer than that between the father and the child. Fathers remain an important parental figure, however, and also influence the process of their children’s development, however, knowledge regarding how fathers should raise their children scientifically and effectively is still developing in China.

## 2. Literature review and development of hypotheses

### 2.1. Fathers’ presence and adolescents’ interpersonal relationship quality

The study of fathers’ presence is a new field in father–child relationships. Krampe (2009) proposed a dynamic theoretical model of fathers’ presence and defined it as psychological closeness and accessibility to their children. Psychological presence suggests that the father is visible and within reach most of the time (Krampe, 2009; Pu et al., 2011). In a broad sense, the interpersonal relationships of teenagers can refer to their social relationships with all the people associated with them. Interpersonal relationship quality is a measure of the quality degree or rank of an individual’s interpersonal relationships, such as high quality or low quality (Seto and Davis, 2021).

Several studies have shown that fathers’ presence influences teenagers’ interpersonal relationship quality. Yoon et al. (2022) suggested that the presence of a father is conducive to the construction of a positive parent–child relationship, thus effectively buffering the adolescent problem behavior. Gleitsmann (2016) concluded that fathers’ presence facilitates the development of father-child relationships. Ying et al. (2021) found that a high paternal presence had obvious advantages for young teenagers’ empathy and sympathy and made it easier to establish harmonious interpersonal relationships. Furthermore, Morales-Castillo (2022) showed that parents’ perceived involvement (PI) had a significant impact on the school performance (SP) of teenagers, and interpersonal relationships were a crucial evaluation standard of SP; therefore, fathers’ parenting also had an impact on teenagers’ interpersonal performance in school. In summary, fathers’ presence was positively associated with adolescents’ interpersonal relationship quality. The higher the quality of father’s presence, the more harmonious interpersonal relationships adolescents will exhibit.

However, in extreme circumstances, such as when fathers are in prison and completely absent from their children’s lives, their roles are restricted, and it is difficult for them to perform their fatherly duties in their children’s daily life, resulting in the absence of education by fathers. This can lead to social problems such as family tension and children’s interpersonal difficulties. Therefore, the introduction of social practice work systems in some countries helped maintain good father–child relationships (Liu C. et al., 2022). Another view is that the harm caused to teenagers’ interpersonal relationships by the absence of fathers can be compensated by education provided by mothers or others (Liu C. C. et al., 2022).

Although a high-quality father’s presence may help promote teenagers’ interpersonal relationships (Palkovitz, 2019), there may not be a direct relationship between fathers’ presence and quality of interpersonal relationships. Previous studies have shown that, when fathers are present, teenagers may experience interpersonal crises (Vass and Haj-Yahia, 2022), while when they are absent, adolescents show good interpersonal relationships (Liu C.-C. et al., 2022). Thus, certain factors regarding fathers’ presence may impact the quality of interpersonal relationships.

Therefore, we proposed the following research hypothesis:

Hypothesis 1: Fathers’ presence positively affects teenagers’ interpersonal relationship quality.

### 2.2. Mediating role of social responsibility in the relationship between fathers’ presence and interpersonal relationship quality

Teenagers’ sense of social responsibility refers to a strong emotional and personality trait of concern for themselves, family, others, the collective, the nation, humanity, and nature, which are influenced by various external things in daily life (Chen, 2013). The understanding of social responsibility is a process of deepening through cognition, perception, and action. From the psychological perspective, the sense of social responsibility refers to a relatively stable psychological quality of individuals who actively undertake social responsibility or help others, which has important practical significance (Huang et al., 2016). Previous studies have shown that the sense of responsibility is an effective predictor of a series of positive psychology characteristics and behaviors, such as job performance, academic achievement, sound development of personality, positive self-evaluation, attitude toward setbacks and failures, and altruistic behavior (Wentzel, 1991; Leyton Román et al., 2019; Otto et al., 2022; Rajasekar et al., 2022; Scholten et al., 2022; Zyuzev, 2022). Scholars have confirmed the influence of fathers’ presence on teenagers’ social responsibility. On the one hand, it has been shown that responsible fathers, who are involved in the development of children as male role models, contribute to the development of a sense of social responsibility in children (Randles, 2020; Donnelly et al., 2022; Kohl et al., 2022). On the other hand, researchers found that absence of fathers may lead to teenagers’ depression, self-loathing, and impulsion and increase their probability of random sexual behavior, whereas casual sex reflected teenagers’ indifference to a sense of responsibility for marriage and love to a certain extent (Liu, 2017; Hehman and Salmon, 2021). Therefore, to reduce the occurrence of this phenomenon and enhance the sense of responsibility of teenagers, the fathers needed to be present.

Among many factors that affect social responsibility, interpersonal relationship quality has become a crucial predictor (Huang et al., 2016), Social responsibility will affect interpersonal relationship quality (Kim and Kim, 2016). Drawing on the theory of social identity (Sewell et al., 2022), in which it is proposed that every member of society belongs to a group, constructing an effective social identity helped mobilize individual social responsibility, further developing harmonious social group relations. Therefore, some scholars have used social identity theory to test the impact of social responsibility on all kinds of social relations. The results showed that the stronger the sense of social responsibility, the more conducive it is to the construction of benign social relations as opposed to bad ones (Grebenshchikova, 2020; Paruzel et al., 2020; Silva et al., 2021; Sorour et al., 2021; Blasco, 2022; Brown et al., 2022).

According to family ecosystem theory, the father-child relationship is influenced by many factors. On the one hand, the father’s relationship with grandfather affects the father-child relationship through intergenerational transmission; on the other hand, the mother has expectations of the father’s image due to the influence of maternal grandfather. The mother’s relationship with maternal grandfather will shape the father’s relationship with her child (Eppler, 2019). Therefore, some studies have shown that the relationship between mother and grandfather, mothers’ support for the father-child relationship, and beliefs about fathers can positively predict the sense of responsibility in marriage and love (Liu, 2017). This shows that while fathers influence teenagers’ interpersonal relationships through social responsibility, the relationship between individuals and others can in turn explain their future sense of responsibility.

Therefore, we proposed the following research hypothesis:

Hypothesis 2: Social responsibility plays an intermediary role in the presence of teenagers’ fathers and quality of interpersonal relationships.

### 2.3. Moderator role of parenting style in the relationship between fathers’ presence and interpersonal relationship quality

Generally, fathers’ presence affects teenagers’ interpersonal relationship to varying degrees, and patterns of father involvement and the quality of father-child relationships tend to be transmitted from generation to generation. Moreover, parenting style plays a key role in determining the quality of transmission (Jessee and Adamsons, 2018). Parenting style can be defined as the method or way of parenting (Azahari and Amir, 2022). Baumrind (1967) divided parenting styles into three types: permissive, authoritative, and authoritarian. “Authoritative” is a democratic parenting style that is more conducive to children’s development, whereas an authoritarian parenting style has adverse effects on children (Masitah and Pasaribu, 2022). Parenting style plays a vital role in the development of teenagers’ social communication ability (Uma and Manikandan, 2022). Some scholars have verified that fathers’ parenting style and attitudes are related to children’s positive biases (Ziv and Arbel, 2021) and adaptive behaviors (Sabat et al., 2021); however, no such correlation was found for mothers. In addition, parental rearing patterns shape college students’ sense of social responsibility by influencing self-efficacy (Shen and Li, 2020). Fathers’ presence promote social responsibility in adolescents, and parenting styles play a moderating role (Liu, 2017; Krohn et al., 2019; Xia et al., 2020; Hehman and Salmon, 2021). Meanwhile, parenting styles play a regulatory role in many fields, such as early puberty and drinking behavior (Ling et al., 2022), perceived father-child facial resemblance and academic performance (Tu et al., 2021), and teenagers’ psychological flexibility (Bibi et al., 2022).

Therefore, we proposed the following research hypothesis:

Hypothesis 3: Parenting styles play a moderating role in the first half of the fathers’ presence on social responsibility and the quality of interpersonal relationships.

## 3. Materials and methods

### 3.1. Participants and procedure

The sample for this study was drawn from May to June 2020, during the COVID-19 outbreak, when school began to resume. This study investigated high school and college students in Hubei Province by random sampling, including a limited number of participants in their 30 s. Our study was approved by the appropriate research ethics committee, and all participants provided written informed consent to participate. A survey was conducted through offline sampling and online completion among senior high school students and college students using the Comprehensive Diagnosis Scale of Interpersonal Relationship, Social Responsibility- Behavior Scale, Ways of Upbringing Scale, and Fathers’ Presence Scale. A total of 1,942 questionnaires were collected, of which 331 showed no effect of fathers on development; thus, the data on fathers’ presence were blank (Because this portion of subjects were left-behind children or from single-mother families, they might not have fathers or their fathers might be absent from home for a long time. Therefore, they basically had no impression of their fathers’ presence during their growing up, their fathers had little influence on them, and they refused to participate in this part of the data survey). The sample details are shown in Table 1.

**TABLE 1:** Basic participant information.

Variable	Group	*N*	Percent (%)	Variable	Group	*n*	Percent (%)
Home address	City	876	45.1	Learning-working status	In high school	915	47.1
	Village	838	43.2		In college	935	48.1
	Suburbs	228	11.7		At work	92	4.7
Gender	Man	760	39.1	Only child	Yes	821	42.3
	Woman	1,182	60.9		No	1,121	57.7
Age (years)	Under 18	922	47.5	Father’s educational level	Primary school or below	222	11.4
	18–24	922	47.5		Junior school	685	35.3
	25–30	76	3.9		High school	587	30.2
	30–40	22	1.1		Junior colleges	187	9.6
					Bachelor or above	261	13.4

*N* = 1,942.

### 3.2. Measures

#### 3.2.1. Comprehensive diagnosis scale of interpersonal relationship

The Comprehensive Diagnosis Scale of Interpersonal Relationship was compiled by Professor Zheng Richang of Beijing Normal University (Adapted from SymptomChecklist90, SCL-90; Cattell, sixteen personality factor questionnaire, 16PF; Eysenck Personality Questionnaire, EPQ, etc.). Interpersonal relationship quality is measured using the Interpersonal Relationship Barriers Scale, which reflects the quality of interpersonal relationships by means of reverse scoring; the higher the score on the scale, the more interpersonal barriers there are, and the worse the relationship is. It evaluates four aspects of the current interpersonal status of participants: talking, making friends, interacting with people, and interacting with the opposite sex (Shi, 2017). There are 28 questions with “yes” or “no” answers; “yes” is recorded as 1 point, and “no” as 0 points. The higher the score, the lower the comprehensive evaluation of interpersonal relationships. The consistency reliability of the questionnaire was 0.89, and the construct validity was 0.67.

#### 3.2.2. Social responsibility-behavior scale

This questionnaire, compiled by Chen (2013), includes cognition, emotion, and action of social responsibility, self-evaluation, school evaluation, and social evaluation dimensions of college students’ social responsibility. The Cronbach’s α coefficient was 0.695, with good reliability, content validity, criterion-related validity, and framework validity. This study selected the self-evaluation dimension of social responsibility for the analysis. The scoring scale of the questionnaire is from 1 to 5, and the scores from low to high indicate “very poor, poor, average, good, very good.” The higher the score, the stronger the social responsibility of the participants (Chen, 2013). In addition, from a psychological point of view, social responsibility includes cognitive, affective, and behavioral responsibility. However, during the measurement process of the pre-survey, we found that the affective dimension of social responsibility carries very large social approbation, which does not truly reflect social responsibility and hardly supports the investigation process of the scale. Thus, we weaken the affective dimension of social responsibility and emphasize the cognitive and behavioral dimensions.

#### 3.2.3. Ways of upbringing scale

This scale contained a multiple-choice question: “What is your fathers’ parenting style?” Options included democratic, authoritative, indifferent, and doting. The multiple-choice question was a category variable. In the data statistics, the democratic parenting style was recorded as 1 and the others were recorded as 0; the category variable was then analyzed.

#### 3.2.4. Fathers’ presence scale

There are 31 entries in the Chinese Short Version of Fathers Questionnaire (FPQ-R), which contains three high-order dimensions (relationship with fathers, family intergenerational relations, beliefs about fathers) and eight subscales (feelings for fathers, mothers’ support for father–child relations, perceptions of fathers’ participation, physical interaction with fathers, parental relations, relationship between mothers and grandfathers, relationship between fathers and grandfathers, and the concept of fathers’ influence). The Cronbach’s α coefficient of internal consistency reliability of the three high-order dimensions and eight subscales of FPQ-R-B was over 0.73, which indicated that all high-order dimensions and their subscales, as well as the items in each subscale, had good consistency and that the scale had certain discrimination validity and calibration correlation validity (Pu et al., 2012; adapted from Father Presence Questionnaire, FPQ, Krampe and Newton, 2006).

## 4. Results

**FIGURE 1:**
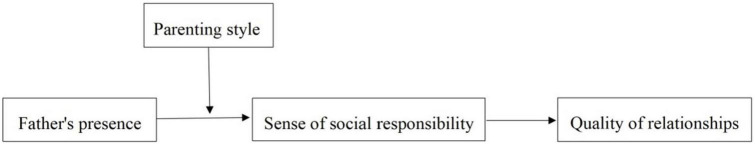
Research model diagram.

**FIGURE 2:**
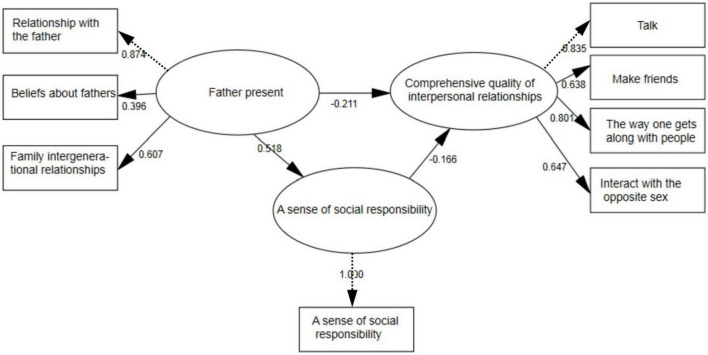
Mediating model of social responsibility.

**FIGURE 3:**
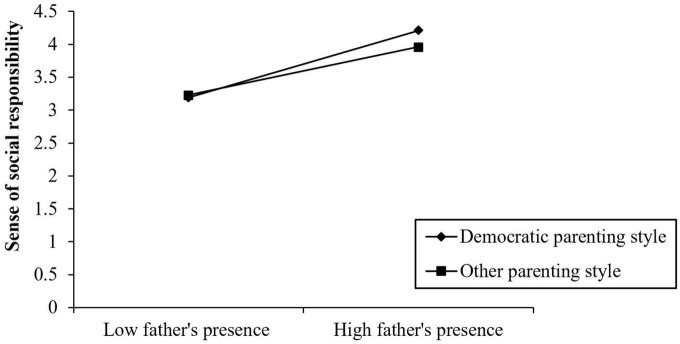
Interactive effect of parenting style and fathers’ presence on sense of social responsibility.

**FIGURE 4:**
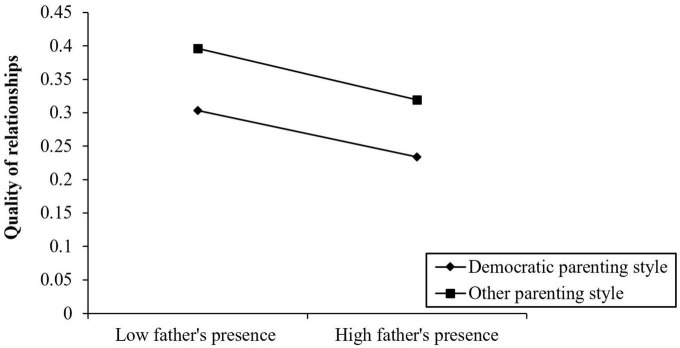
Interactive effect of parenting style and fathers’ presence on quality of relationships.

This study used PROCESS macro of SPSS 24.0 and Amos 26.0 for data management and analysis. First, SPSS 26.0 was used to process subject base information (see Table 1); combining overall description of each variable (see Table 2); getting analysis of the correlation between variables (see Table 3). Second, AMOS was used to calculate the fit index of the mediation model (see Table 4); it was used to construct structural equation modeling and test the mediating effect of social responsibility. Thereafter, the fitted indicators are shown in Table 4, and the mediating effect between variables can be shown in Figure 1. The magnitude of the effect between the variables can be shown in Figure 1 (see Figure 1). Next, PROCESS was used to build “research model diagram” (see Figure 2), to measure the “interactive effect of parenting style and fathers’ presence on quality of relationships” and “interactive effect of parenting style and fathers’ presence on quality of relationships”e(see Figures 3, 4). Finally, PROCESS and AMOS were applied to test fitting indicators of the adjustment model (see Table 5).

**TABLE 2:** Overall situation of fathers’ presence, social responsibility, and comprehensive quality of interpersonal relationships.

Inventory	*n*	Min	Max	*M*	SD	Theoretical median
Social responsibility	1,941	1	5	3.57	0.71	3
Fathers’ presence	1,285	1.5	5	3.51	0.71	3
Comprehensive quality of interpersonal relationship	1,942	0	1	0.32	0.20	0.5

*N* = 1,942.

**TABLE 3:** Correlation analysis of fathers’ presence, social responsibility, and comprehensive quality of interpersonal relationship.

	1	2	3	4	5	6	7	8
1. Sense of social responsibility	–							
2. Relationship with father	0.44***	–						
3. Family intergenerational relationships	0.34***	0.53***	–					
4. Beliefs about fathers	0.24***	0.36***	0.20***	–				
5. Conversation	−0.23***	−0.22***	−0.18***	−0.23	–			
6. Treatment of people and things	−0.19***	−0.21***	−0.13***	−0.37	0.69***	–		
7. Making friends	−0.16***	−0.13***	−0.13***	0.33	0.57***	0.54***	–	
8. Associating with the opposite sex	−0.16***	−0.15***	−0.12***	0.17	0.57***	0.57***	0.48***	–
M	3.57	3.40	4.00	3.04	0.35	0.48	0.19	0.26
SD	0.71	0.90	0.70	1.13	0.27	0.31	0.21	0.25

*N* = 1,942. *P < 0.05, **P < 0.01, and ***P < 0.001, the same below.

**TABLE 4:** Structural equation model fitting table (*N* = 1,942).

Category	χ^2^/df	GFI	AGFI	NFI	CFI	RMSEA
Threshold	<3	>0.9	>0.9	>0.9	>0.9	<0.05 good; 0.05–0.10 moderate;>0.1 bad
Mediated model	2.865	0.990	0.980	0.982	0.988	0.038

**TABLE 5:** Hierarchical linear regression analysis results.

Variable	First stage (Dependent: Sense of social responsibility)						Second stage (Dependent: Quality of relationships)					
	First step			Second step			Third step			Fourth step		
	β	SE	*t*	β	SE	*t*	β	SE	*t*	β	SE	*T*
Constant	0.03	0.03	1.01	0.01	0.04	0.36						
FP	0.41***	0.03	16.68	0.33	0.05	6.02						
PS	0.06	0.04	1.43	0.07	0.04	1.70						
FP*PS				0.13**	0.06	2.06						
Constant							0.17**	0.05	3.45	0.16**	0.06	2.79
FP							−0.06	0.04	−1.40	−0.09	0.08	−1.05
PS							−0.31***	0.06	−5.29	−0.30***	0.07	−4.64
SSR							−0.29***	0.04	−7.11	−0.29***	0.05	−6.32
FP*PS										0.05	0.09	0.50
*R* ^2^	0.19			0.19			0.09			0.09		
Adj*R*^2^	0.19			0.19			0.08			0.08		
*F*	150.45***			102.60***			39.44***			29.65***		
Δ*R*^2^				0.002						0.000		

FP, fathers’ presence; PS, parenting style; SSR, sense of social responsibility. *N* = 1,942. *P < 0.05, **P < 0.01, and ***P < 0.001, the same below.

### 4.1. The current situation of fathers’ presence, social responsibility, and interpersonal relationship quality

As shown in Table 2, the average social responsibility of teenagers was 3.57. Taking the theoretical median value of 3 as the reference point, the single sample *T*-test showed a significant difference between the scores and median values of teenagers’ social responsibility. This indicated that teenagers’ sense of social responsibility was generally higher than the theoretical median level and was generally at the upper-middle level. Moreover, the standard deviation of 0. 73 indicated that the dispersion of adolescents’ sense of social responsibility was greater; that is, the level of adolescents’ sense of social responsibility showed large variations, which indicated that teenagers’ sense of social responsibility had large individual differences. In addition, among the three factors of fathers’ presence, the score for family intergenerational relationships was the highest, the score for relationship with father was the second highest, and the average score for beliefs about one’s father was the lowest. Regarding the comprehensive quality of interpersonal relationships, the overall mean value is 0.32, which is better than medium. The results of the single-sample *T*-test showed that the comprehensive quality of teenagers’ interpersonal relationships was significantly lower than the theoretical average. Among the four dimensions, the highest-ranked dimension was making friends, followed by heterosexual communication, conversation, and treatment of people and things. Among the types of relationships with fathers, 68.2% of participants reported that they had democratic father–child relationships, and other types of father–child relationships accounted for 31.8%.

### 4.2. Correlation analysis of fathers’ presence, social responsibility, and interpersonal relationship quality

According to Pearson correlation coefficient analysis, there was a pairwise correlation between adolescents’ fathers’ presence, social responsibility, and interpersonal relationship quality. The presence of teenagers’ fathers was positively correlated with social responsibility and negatively correlated with the quality of interpersonal relationships, with the correlation coefficients, r, being 0.44 and −0.16, respectively. Moreover, there was a significant negative correlation between social responsibility and interpersonal relationship quality, and the correlation coefficient r was −0.23 (*p* < 0.001).

#### 4.2.1. Correlation analysis of fathers’ presence, social responsibility, and interpersonal relationship quality in different dimensions

There was a significant positive correlation between the dimensions of teenagers’ fathers’ presence and social responsibility, and the correlation coefficient was between 0.235 and 0.437. Among them, the correlation coefficient between the dimension of the relationship between teenagers and their fathers and their sense of social responsibility was higher. There was a significant negative correlation in fathers’ presence between the dimensions of “adolescent fathers’ relationship with their fathers,” family intergenerational relationship, and the dimension of the comprehensive quality of interpersonal relationships. And the correlation coefficient was between −0.121 and −0.218. Adolescents’ sense of social responsibility was negatively correlated with all dimensions of interpersonal relationship quality, and the correlation coefficient was between −0.158 and 0.234.

There was a close relationship between the teenagers’ fathers’ presence, social responsibility, and interpersonal quality.

#### 4.2.2. Mediating analysis of social responsibility in the influence of fathers’ presence on interpersonal relationship quality

The above conclusion shows a significant correlation between teenagers’ fathers’ presence, social responsibility, and interpersonal quality, which aligns with the test premise of intermediary utility. To identify how teenagers’ sense of social responsibility mediated the relationship between fathers’ presence and interpersonal relationship quality, this study adopted the structural equation model. In this study, chi-squared ratio degrees of freedom (χ^2^/df), Root–Mean–Square Error of Approximation, Goodness-of-Fit Index, Adjusted Goodness-of-Fit Index, Normed Fit Index, and Comparative Fit Index were all in line with the structural equation fit standards (Table 4).

When fathers’ presence affected the sense of social responsibility, the standardized path coefficient value was 0.518 > 0, and this path showed the significance of 0.01 level (*z* = 14.463; *p* = 0.000 < 0.01), thus indicating that fathers’ presence had a significant positive influence on the sense of social responsibility. When the father was present, the normalized path coefficient value was −0.211 < 0, and this path showed a significant level of 0.01 (*z* = −5.108; *p* = 0.000 < 0.01), which demonstrated that the fathers’ presence had a significant negative impact on the overall quality of interpersonal relationships. When social responsibility affected the overall quality of interpersonal relationships, the standardized path coefficient was 0.166 < 0, and this path showed a significance level of 0.01 (*z* = −4.621; *p* = 0.000 < 0.01), indicating that social responsibility had a significant negative impact on the overall quality of interpersonal relationships.

Teenagers’ fathers’ presence significantly positively predicted their social responsibility (β = 0.435, *P* < 0.001) and negatively predicted their interpersonal relationship quality (β = −0.068, *P* < 0.05), and adolescents’ sense of social responsibility significantly negatively predicted the quality of their interpersonal relationships (β = −0. 217, *P* < 0.001). After analyzing social responsibility as an intermediary variable, the negative predictive effect of fathers’ presence on interpersonal relationship quality reached a significant level (β = −0. 211, *P* < 0.001), which indicated that social responsibility played a partial intermediary role between adolescents’ fathers’ presence and the comprehensive quality of interpersonal relationships, and the intermediary effect was 0.518*0.166/(0.211 + 0.518*0.166) = 29.0%. The mediating role of social responsibility was obvious. The degree of fathers’ presence enhanced the social responsibility of teenagers. Teenagers with higher social responsibility had better interpersonal relationship quality.

#### 4.2.3. The moderating effect of fathers’ parenting style in social responsibility on fathers’ presence and interpersonal relationship

First, fathers’ presence, social responsibility, interpersonal quality, and parenting style were standardized, and the parenting style was multiplied by fathers’ presence to form a product term. The hierarchical linear regression was then used to test the hypothesis of the adjustment effect of parenting style. The analysis results of the hierarchical linear regression are shown in Table 5.

In Equation 1, the regression coefficient of fathers’ presence was significant (β = 0.413, *P* < 0.001), which indicated that fathers’ presence positively affects social responsibility. In Equation 2, the interaction between fathers’ presence and parenting style had a positive predictive effect on social responsibility (β = 0.218, *P* < 0.05), and the moderating effect was Δ*R*^2^ = 0.002. At this time, the independent effect of fathers’ presence on social responsibility disappeared. In Equations 3 and 4, fathers’ parenting style directly affected the quality of teenagers’ interpersonal relationships, and a democratic father–child relationship optimized the quality of teenagers’ interpersonal relationships, however, parenting style regulated the first half of the intermediary process “fathers’ presence → social responsibility → interpersonal relationship quality,” that is, the influence of fathers’ presence on teenagers’ social responsibility was related to fathers’ parenting style. If fathers adopted a democratic parenting style, their presence directly promoted children’s social responsibility; if fathers adopted other parenting styles, the influence of fathers’ presence on children’s responsibility was greatly weakened.

According to the test method of intermediary moderating variables put forward by Wen et al. (2006), the regression of parenting style and fathers’ presence on interpersonal relationship quality was not significant, and the moderating effect of parenting style was entirely through the intermediary variable of social responsibility. To explain the moderating effect, we set the regression return of social responsibility and interpersonal relationship quality to, respectively, predicted variables as follows:

Social responsibility = 0.128 fathers’ presence*parenting style.

Quality of interpersonal relationship = 0.163–0.30 parenting style −0.291 sense of social responsibility = 0.163–0.30 parenting style −0.291*0.128 fathers’ presence*parenting style.

## 5. Discussion

Most previous studies focused on the effects of fathers’ presence on adolescent development, but rarely examined the mechanisms underlying the presence of fathers on adolescent development. Moreover, previous studies ignored the impact of fathers’ way of being present on adolescent interpersonal relationships. Based on social identity theory, the present study introduced adolescents’ social responsibility as a mediating variable to explore the influence of father’s presence style on adolescents’ interpersonal. Our results showed that fathers’ presence was positively related to relationships, but only for participants who adopted democratic parenting styles. This finding corroborates the results of previous studies. Further, we investigated the current situation of fathers’ presence, social responsibility, and interpersonal relationship quality and provided the influence mechanism of fathers’ presence on interpersonal relations; that is, fathers’ presence significantly affected the quality of interpersonal relationships among adolescents by influencing social responsibility, the democratic parenting method played a regulatory role, and the process included a regulated intermediary effect. The findings of this study enrich the literature by exploring the significance of emphasizing fathers’ democratic presence on teenagers’ sense of social responsibility and interpersonal relationships. The practical implications of this study are that society should encourage more fathers to be present and guide them to adopt a democratic parenting style that will benefit adolescents’ development and family wellbeing.

### 5.1. Theoretical contributions

This study has important implications for both theory and practice. From a theoretical perspective, our results provide the mechanism of influence of fathers’ way of being present on teenagers’ sense of social responsibility and interpersonal relationships. Fathers’ democratic presence had a far-reaching impact on teenagers’ sense of social responsibility and the quality of their interpersonal relationships. Kim et al. (2021) studied the parent–child relationships of adolescents in South Africa, emphasizing the importance of fathers in reducing the risk of HIV and drinking behavior in adolescent children. Rivers et al. (2022) elaborated the interactive relationship between patrilineal attachment and anxiety and depression in high-risk adolescents from the perspective of attachment theory. O’Gara et al. (2022) described the unique role of fathers in reducing their daughters’ suicidal ideation and trying risky behaviors.

First, fathers’ presence includes three high-order dimensions: the highest dimension is that for family intergenerational relationships, which includes the relationship between mother and grandfather and that between father and grandfather; relationship with father takes second place, and the father’s influence is the lowest dimension. Research shows that during the growth of teenagers, fathers’ presence is not looking good, and its important role is seriously underestimated. Second, according to attachment theory and Paquette’s “activation” relationship theory (Paquette et al., 2000), the most basic relationship formed by children is first the mother–child relationship, then the father–child relationship, and social relationships develop later; furthermore, fathers’ presence has a significant impact on their children’s sense of social responsibility, especially when they interact with the general community. Finally, fathers’ presence can promote the quality of teenagers’ interpersonal relationships; in other words, the better the relationship with the father, the higher the quality of interpersonal relationships. Existing research has found the mechanism of the influence of fathers’ presence on relationships. Fathers’ participation in parenting had a buffering effect on mother’s negative parenting, and the buffering effect of fathers’ participation in parenting time was particularly obvious (Liu et al., 2013). This study found that parenting style moderated the first half of the pathway of the mediating effect of fathers’ presence on the effect of social responsibility on interpersonal relationship quality.

Undemocratic parenting styles tend to counteract the effect of father’s presence on adolescents’ sense of social responsibility. In turn, this also reduces the role of father’s presence in adolescents’ interpersonal interactions. In contrast, democratic parenting not only promotes a greater sense of social responsibility among adolescents but also significantly enhances their interpersonal skills. Attachment theory, object relation theory, intrinsic fatherhood theory, and analytical psychology theory all posit that children seek their fathers as an innate attribute (Pu et al., 2017).

### 5.2. Practical implications

According to a White Paper on the *status quo* of Family Education in China, the data on fathers’ presence show that less than 20% of children’s family education is dominated by fathers (Jiang, 2018). An investigation on the quality of teenagers’ interpersonal relationships finds that what they are most worried about is the dimension of “the way one gets along with people,” which is manifested in unnatural communication with strangers, nervousness, embarrassment, worry, lack of confidence, and so on. The phenomenon that fathers’ presence is not viewed positively and that their roles are underestimated and negatively judged in the development of adolescents urgently needs to be ameliorated, because it is not conducive to adolescent development.

Adolescents’ social responsibility is significantly positively correlated across all three dimensions of father’s presence, implying that the quality of father’s presence enhances adolescents’ sense of social responsibility. To enhance adolescents’ sense of social responsibility, we need to encourage them to participate more in social practices and enhance their awareness of group service; however, fathers should fully realize their potential and role and attend to their words and deeds during their children’s growth, setting a good example. A good father–child relationship can help children face danger, resist pressure, and dare to survive independently (Yogman and Eppel, 2022). When the father is able to respond to his children in exciting and challenging interactive ways with games (neither too boring nor too scary, but appropriately excited), we can predict that his children will have high social functioning at the ages of 16–22.

If there is sufficient companionship and instruction from the father in the process of growing up, and teenagers receive four aspects of guidance—talking to others, dealing with others, making friends, and interacting with the opposite sex—they will be more confident in interpersonal communication, have a stronger sense of social responsibility, and have a higher quality of interpersonal relationships (Zhu, 2016). Interpersonal responsibility may be a cross-situational personality trait that can be cultivated in three spheres: family, school, and society (He and Huang, 2017). Therefore, improving teenagers’ sense of social responsibility can also promote the quality of their interpersonal relationships.

Based on the above conclusions, fathers’ presence and social responsibility have a significant impact on the quality of interpersonal relationships among teenagers. Society should encourage fathers to be more present, and a democratic upbringing is beneficial to the establishment of teenagers’ sense of social responsibility and the improvement of the quality of their interpersonal relationships. In addition, fathers’ participation in children’s early life, games, and activities and in raising and educating children plays a decisive role in children’s psychological development and social adaptation (Wang et al., 2012). Fathers’ positive presence should be based on a harmonious father–child relationship (Hewlett, 2000). Democratic father–child relationships, the idea that fathers and mothers are equally important in the growth of their children (Grossmann et al., 2002), and the idea that fathers should participate in the upbringing of their children need to permeate all aspects of social life, such as schools, hospitals, government agencies, laws and social policies. The recommendations of this paper to the Chinese government, based on the findings of the study, are to improve the Family Education Promotion Act (implemented 1 January 2022), understand the need for fathers to participate in parenting, and implement appropriate guarantee mechanisms. This should encourage fathers to adopt a scientific approach to their children’s upbringing. Before participating in parenting, fathers, as well as mothers, should receive professional training and learn scientific parenting models, which is conducive to child development and family wellbeing.

### 5.3. Limitations and directions for future research

This study had several limitations.

First, the sample size of this study is not sufficient. Our sample was mainly collected from Wuhan, Hubei, and although Wuhan is geographically located in central China, with a medium level of economic development, whether it is representative of the national average level of fathers’ presence in parenting is open to be discussed. Therefore, in subsequent studies, researchers can collect data from the whole country, making the findings more generalizable.

Second, the age group coverage of the subjects in this study is not comprehensive enough. Our research subjects are mainly adolescents; the adolescent period is an important stage in a person’s life, with physical and intellectual abilities at the peak of life, and is highly researchable. However, other stages of a person’s life are also important, such as early childhood, which is the period when an individual’s brain is developing fastest and social skills are developing optimally (Uchitel et al., 2022). If fathers are present during this period and administer appropriate early education in the right way to their young children, it is likely that the young children will be better socialized in the future. Therefore, future research could explore the effects of father’s presence styles on other aspects of individuals at different ages, leading to more interesting conclusions.

Third, the influence of fathers’ presence and social responsibility on the quality of adolescents’ interpersonal relationships may be the result of a synergistic effect of multiple factors. The discussion did not fully explain the reasons behind the influence of father’s presence and responsibility on the quality of adolescent interpersonal relations. Is it the growth of teenagers themselves or the family and social environment, or is it the result of joint action? Since our article focuses on the presence of fathers and their role as role models, we do not go further into the reasons behind the findings.

Finally, there was no follow-up study conducted as part of this research. Although our study made feasible recommendations, it remains to be explored whether the quality of fathers’ presence for the participating adolescents improved. Therefore, in future studies, researchers can conduct systematic, regular studies on the same cohort of respondents over a relatively long period of time (i.e., longitudinal research) to understand the implementation of countermeasures, provide feedback, and modify and improve countermeasures.

## Data availability statement

The raw data supporting the conclusions of this article will be made available by the authors, without undue reservation.

## Ethics statement

The studies involving human participants were reviewed and approved by the Institutional Review Board at Hubei University of Education of China. Written informed consent to participate in this study was provided by the participants’ legal guardian/next of kin.

## Author contributions

AL and LS conceived and designed the study and collected the data. AL and SF analyzed and interpreted the data, wrote the manuscript, and were responsible for funding the acquisition. AL, LS, and SF contributed to the revision process of the manuscript. All authors contributed to the article and approved the submitted version.
